# The Role of DNA Damage in Neural Plasticity in Physiology and Neurodegeneration

**DOI:** 10.3389/fncel.2022.836885

**Published:** 2022-06-23

**Authors:** Anna Konopka, Julie D. Atkin

**Affiliations:** ^1^Centre for Motor Neuron Disease Research, Macquarie Medical School, Faculty of Medicine, Health and Human Sciences, Macquarie University, Sydney, NSW, Australia; ^2^La Trobe Institute for Molecular Science, La Trobe University, Melbourne, VIC, Australia

**Keywords:** synaptic plasticity, neural plasticity, DNA damage, DNA repair, neurodegeneration

## Abstract

Damage to DNA is generally considered to be a harmful process associated with aging and aging-related disorders such as neurodegenerative diseases that involve the selective death of specific groups of neurons. However, recent studies have provided evidence that DNA damage and its subsequent repair are important processes in the physiology and normal function of neurons. Neurons are unique cells that form new neural connections throughout life by growth and re-organisation in response to various stimuli. This “plasticity” is essential for cognitive processes such as learning and memory as well as brain development, sensorial training, and recovery from brain lesions. Interestingly, recent evidence has suggested that the formation of double strand breaks (DSBs) in DNA, the most toxic form of damage, is a physiological process that modifies gene expression during normal brain activity. Together with subsequent DNA repair, this is thought to underlie neural plasticity and thus control neuronal function. Interestingly, neurodegenerative diseases such as Alzheimer’s disease, amyotrophic lateral sclerosis, frontotemporal dementia, and Huntington’s disease, manifest by a decline in cognitive functions, which are governed by plasticity. This suggests that DNA damage and DNA repair processes that normally function in neural plasticity may contribute to neurodegeneration. In this review, we summarize current understanding about the relationship between DNA damage and neural plasticity in physiological conditions, as well as in the pathophysiology of neurodegenerative diseases.

## Introduction

DNA damage is now widely implicated in aging and the pathophysiology of age-related neurodegenerative disorders, such as amyotrophic lateral sclerosis (ALS), Alzheimer’s disease (AD), Huntington’s disease (HD), and Parkinson’s disease (PD) (Madabhushi et al., 2014; Machiela and Southwell, 2020). However, emerging evidence suggests that DNA damage and DNA repair are not only induced by pathological conditions (Ju et al., 2006; Tiwari et al., 2012; Suberbielle et al., 2013; Madabhushi et al., 2015). The same processes involved in neurodegeneration as we age are also involved in fundamental physiological functions of neurons that are related to neural plasticity. Hence, DNA damage and repair are associated with neural plasticity, implying an important role for these processes in neuronal function. Furthermore, in neurodegenerative diseases the selective death of specific groups of neurons is present. This suggests that the unique properties of neurons may contribute to selective neurodegeneration in pathophysiology. There are no previous comprehensive reviews on the role of DNA damage and repair in neural plasticity in neurodegenerative disorders. A previous collection entitled “DNA Damage, Neurodegeneration, and Synaptic Plasticity” (consisting of three research articles and one review) covered only some aspects of this topic (Merlo et al., 2016). In this review, we summarize current knowledge regarding the link between neural plasticity and DNA damage in both physiological conditions and in the pathophysiology of neurodegenerative diseases.

## Types of DNA Repair in Neurons

DNA is subject to persistent assault from both endogenous and environmental sources, and specific molecular pathways detect and respond to specific types of damage (Jackson and Bartek, 2009). These mechanisms, collectively known as the “DNA damage response (DDR)”, are essential for neuronal viability, although they decline significantly during aging (Agathangelou et al., 2018). DNA can be damaged in several different ways. When the phosphodiester bonds break in one or both DNA strands, this results in single-stranded and double stranded breaks (SSBs and DSBs respectively). Whilst DSBs arise less frequently than SSBs, they are much more harmful (Chatterjee and Walker, 2017). Each type of damage requires a specific mechanism of DNA repair, although these processes can overlap (Ma and Dai, 2018).

Neurons are terminally differentiated, post-mitotic, non-replicating, long-lived cells, with high metabolic activity. Therefore, their ability to cope with DNA damage differs from mitotic or cycling cells because they need to withstand damage throughout their lifespan. Neurons rely mostly on base excision repair (BER) and nucleotide excision repair (NER) pathways to deal with SSBs, and non-homologous end joining (NHEJ) to repair DSBs (Fishel et al., 2007). Here we discuss those DNA repair mechanisms most relevant to neurons. The reader is directed to several excellent recent reviews for a comprehensive discussion of all DNA repair mechanisms^1^ (see DNA damage repair (Internet), 2021).

BER repairs minor base lesions that are not helix distorting, such as the formation of 8-oxo-guanine (8-oxoG) bases resulting from reactive oxygen species (ROS). Following damage, DNA glycosylases such as 8-oxoguanine DNA glycosylase (OGG1) or nei like DNA glycosylase 1 (NEIL1) remove the damaged base, leaving an abasic site. Then, a nick in the phosphodiesterase backbone is made by AP endonuclease 1 (APE1) or a bifunctional glycosylase with lyase activity, creating DNA SSBs. Poly (ADP-ribose) polymerase 1 (PARP1) then senses the SSBs, which undergo repair by short-patch or long-patch BER (Kim and Wilson, 2012).

NER repairs bulky lesions, including cyclobutane-pyrimidine dimers, 6-4 pyrimidine-pyrimidone photoproducts (6-4PPs), chemical adducts, intrastrand crosslinks, and ROS-generated cyclopurines (Horowitz et al., 2011; Gonzalez-Hunt and Sanders, 2021). These lesions are induced mostly by UV radiation and exposure to chemicals that cannot cross the blood-brain barrier. Therefore, the number of sources that can produce these lesions in the brain is limited, although dopamine neurons are vulnerable to oxidative DNA damage which can be repaired by NER (Horowitz et al., 2011; Gonzalez-Hunt and Sanders, 2021). In NER, after the detection of SSBs, an endonuclease complex cuts the damaged strand, followed by gap-filling synthesis and ligation (Chatterjee and Walker, 2017).

NHEJ is the primary DNA repair pathway for DSBs in neurons. DSBs are recognized by ataxia telangiectasia mutated kinase (ATM), which phosphorylates histone H2AX (γH2AX) over the surrounding mega-base region of DNA (Thompson and Limoli, 2000). The broken DNA ends are bound by the DNA protein kinase complex (DNA-PK), which is composed of Ku70/Ku80 heterodimers and the catalytic subunit of DNA-PK (DNA-PKcs; Ciccia and Elledge, 2010). These ends are then processed by the nuclease Artemis and DNA polymerases Polμ or Polλ (Lieber, 2010), and ligated by a DNA ligase complex, containing DNA ligase 4, X-ray cross-complementation group 4 (XRCC4), and XRCC4 like factor (XLF)/Cernunnos (Ahnesorg et al., 2006; Buck et al., 2006). However, when classical NHEJ repair is defective, DSBs can be repaired by alternative end joining repair, A-NHEJ. A subset of A-NHEJ, which relies on micro-homologous sequences on either side of the DSB, is termed microhomology-mediated end joining (MMEJ; Wang and Xu, 2017). MMEJ is an error-prone DSB repair mechanism that results in chromosome translocations and rearrangements (Wang and Xu, 2017). It begins with resection of the broken DNA ends, which facilitates exposure of the micro-homologous sequences that will be ligated. These sequences are then annealed prior to ligation to form an intermediate structure with a 3’-flap and gaps on both sides of the DSB. Subsequently, this intermediate is removed to allow DNA polymerase to fill in the gap and the broken ends are ligated by DNA ligase III/I (Wang and Xu, 2017).

As neurons are post-mitotic, and thus non-replicating, it is believed that DNA repair pathways associated with replicating cells, such as mismatch repair (MMR) and homologous recombination (HR), are absent in neurons (Fishel et al., 2007; Welty et al., 2018). MMR corrects spontaneous base-base mismatches and small insertion-deletion loops (indels) that are generated during DNA replication (Pecina-Slaus et al., 2020). HR requires undamaged DNA on the sister chromatid as a template for repair, therefore it operates mostly during the S and G2 phases of the cell cycle (Welty et al., 2018). However, recent studies have demonstrated that HR may be active in neurons because it can utilize nascent mRNA during active transcription as a template to repair DNA (Fishel et al., 2007; Welty et al., 2018), implying that this mechanism is present in non-dividing, differentiated neurons (Welty et al., 2018).

## Neural Plasticity

Neurons are structurally and functionally unique, with their unusual morphology and their ability to communicate electrically and chemically *via* intricate synaptic contacts. Synaptic plasticity refers specifically to the functional and structural alterations of synapses that modulate the strength and efficiency of communication between neurons. Neural plasticity has a broader meaning and describes the ability of the nervous system to change its activity in response to intrinsic or extrinsic stimuli, by reorganizing its structure, function, or connections. The phenomenon of neural and synaptic plasticity underlies the molecular mechanisms involved in cognitive processes such as learning and memory, but it is also important for brain development and homeostasis, sensorial training, and recovery from brain lesions (Mateos-Aparicio and Rodriguez-Moreno, 2019). Therefore, it is not surprising that disrupted plasticity leads to a decline in cognitive functions and is associated with age-related diseases, including Alzheimer’s disease, amyotrophic lateral sclerosis, Huntington’s disease and Parkinson’s disease (Phukan et al., 2007; Paulsen, 2011; Picconi et al., 2012; Zhao et al., 2014).

Neuronal electrical excitation modulates plasticity (Hogan et al., 2020) by three fundamental mechanisms: (a) long-term depression (LTD), an activity-dependent reduction in the efficacy of neuronal synapses (Ahn et al., 1999); (b) long-term potentiation (LTP), persistent strengthening of synapses that leads to a long-lasting increase in signal transmission between neurons (Kandel, 2001); and (c) activity-associated development of corticospinal circuitry for movement control (Martin, 2005). In parallel with activity-associated plasticity, structural modifications of axonal, dendritic branches, and spine morphology occurs, constituting structural synaptic plasticity (Mateos-Aparicio and Rodriguez-Moreno, 2019).

In the brain, glutamate is the major excitatory neurotransmitter, whereas gamma aminobutyric acid (GABA) is the principal inhibitory neurotransmitter. Both glutamatergic excitatory and GABAergic transmission are important molecular processes involved in synaptic plasticity and neuronal activation. Glutamate receptors mediate fast excitatory synaptic transmission in the CNS, and they regulate a broad spectrum of processes. Ionotropic glutamate receptors are named after their specific ligands: kainate, α-amino-3-hydroxy-5-methyl-isoxazole-4-propionate (AMPA), and N-methyl-D-aspartate (NMDA). The NMDA receptors (NMDARs) are crucial for activity-dependent synaptic changes and learning and memory (Baez et al., 2018). It is also increasingly recognized that NMDARs participate in dendritic synaptic integration and are critical for generating persistent activity of neural assemblies (Hunt and Castillo, 2012). GABAergic synapses, similar to glutamatergic synapses, adjust their strength depending on the pattern of neuronal activity. The plasticity of inhibitory synapses is largely mediated by modulation of the expression, localization, and function of GABA receptors (Mele et al., 2016).

## Neuronal Activation Induces DNA Damage

Several studies have shown that neuronal activity generates DSBs in cultured neurons. Activation of ionotropic NMDA or AMPA/kainate glutamate receptors induced DNA damage in rat cortical neuronal cultures (Crowe et al., 2006). This was detected by the formation of _γ_H2AX foci, implying that DSBs were generated by neuronal activation. Furthermore, this was associated with activation of the Mre11-dependent DNA repair pathway, which functions in DSB repair (Crowe et al., 2006). The induction of DNA damage was related to increases in intracellular calcium levels (Crowe et al., 2006), which is known to contribute to mitochondrial dysfunction and an increase in ROS (Krieger and Duchen, 2002). However, treatment with the antioxidant, Vitamin E, and intracellular calcium chelator, BAPTA-AM, did not protect neurons completely from DNA damage, suggesting that it is also generated by other sources not related to mitochondrial dysfunction and ROS (Crowe et al., 2006).

Similarly, another study showed that cerebral cortical neurons efficiently repair oxidative DNA lesions after transient activation of glutamate receptors (Yang et al., 2010). This was facilitated by BER involving DNA glycosylases OGG1 and NEIL1 and cAMP-response element-binding protein (CREB)-mediated APE1 expression (Stetler et al., 2010; Yang et al., 2010). Furthermore, glutamate also activates nuclear factor-kappa B (NF-kB) in neurons (Jiang et al., 2003), which promotes DNA repair (Wang et al., 2009). Interestingly, elevated levels of intracellular oxidative damage stimulate BER, resulting in an increase in cellular survival (Chen et al., 1998; Ramana et al., 1998; Cabelof et al., 2002). Higher concentrations of glutamate (100 μM) can be deleterious for neurons (Mattson et al., 1988). Interestingly, during learning and memory in hippocampal neurons or activation of motor system neurons during exercise, glutamate concentration in the synaptic cleft transiently increases above 100 μM and reaches millimolar values (Rao-Mirotznik et al., 1998; Choi et al., 2000). Therefore, activation of synaptic glutamate receptors may upregulate DNA repair systems, thereby increasing repair capability in neurons (Yang et al., 2011). However, with a compromised DNA repair system or increased oxidative stress, neurons become rapidly overwhelmed and prone to cell death (Yang et al., 2011).

A recent study concluded that DSBs are generated physiologically to resolve topological limitations to gene expression in neurons. Topoisomerase enzymes participate in the overwinding or underwinding of DNA and thus they manage DNA topological constraints (McKinnon, 2016). Neuronal activity produces DSBs at specific loci *in vitro* by topoisomerase IIβ (TopIIβ), in the promoters of early response genes (ERGs, also called immediate early genes, IEGs) that are crucial for experience-driven changes to synapses, learning, and memory (Ju et al., 2006; Tiwari et al., 2012; Madabhushi et al., 2015). Interestingly, the expression patterns of ERGs in response to neuronal stimulation correlated well with the formation and repair of activity-induced DSBs (Madabhushi et al., 2015), implying that generation of DSBs and their subsequent repair are essential steps for proper gene function. Furthermore, DSBs produced during neuronal excitation were repaired within 2 h of the initial stimulus, suggesting that this process employs rapid DNA repair mechanisms such as NHEJ (Madabhushi et al., 2015). Similarly, in a more recent study, physiological neuronal activity by exposure of mice to learning behaviors through contextual fear conditioning (CFC) induced the formation of DSBs. This was also associated with experience-driven transcriptional changes within neurons (Stott et al., 2021). Similarly, in another recent study, neurons were found to accumulate DNA SSBs at specific sites within the genome. These were repaired by PARP1 and XRCC1-dependent mechanisms and were located within enhancers at, or near, CpG dinucleotides or at sites of DNA demethylation (Wu et al., 2021). Hence, together these studies suggest that DNA damage may facilitate important physiological roles in neurons. This is highlighted by the specific location of the associated DNA breaks, the requirement for gene expression, and their generation as a result of normal physiological processes.

## DNA Damage Modulates Glutamatergic Neurotransmission and Plasticity

Whilst neuronal activation is associated with induction of DNA damage, conversely, DNA damage has been shown to modulate neuronal activity. Interestingly, DNA damage can alter the activity of AMPA glutamate receptors (Lu et al., 2001). Induction of SSBs in neurons with camptothecin, a topoisomerase I inhibitor, resulted in decreases in AMPA-induced current and calcium response to AMPA in whole cell patch clamp analyses. This effect was abolished by pharmacological inhibition of caspases, implying that caspases are involved in this process (Lu et al., 2001). A link between DNA damage and altered glutamatergic neurotransmission induced by stress and anxiety has also been suggested (Reus et al., 2015). Rats selectively bred for an anxiety phenotype, by either high or low contextual fear conditioning, displayed more SSBs and DSBs in the hippocampus, amygdala, and nucleus accumbens compared to control animals. This correlated with changes in expression of NMDA receptor subunits NR1, NR2A, and NR2B, and excitatory amino acid transporter 1 (EAAT1), which removes glutamate from the extracellular space. Expression of NMDA receptor subunits and EAAT1 underwent further alterations when animals were additionally stressed, further linking the anxious phenotype, DNA damage, and glutamatergic system (Reus et al., 2015).

The base-excision DNA repair (BER) pathway has also been linked to maintenance of synaptic plasticity (Yu et al., 2015). Epigenetic modifications play important roles in neuronal plasticity, learning, and memory, and in neurological disorders. Cytosine methylation is the major covalent modification of eukaryotic genomic DNA and demethylation is mediated by the ten-eleven translocation (Tet) family of proteins. Interestingly, Tet-initiated DNA demethylation is mediated through BER in neurons (Yu et al., 2015) and synaptic activity bi-directionally regulates neuronal expression of Tet3, and hence DNA demethylation through BER. Expression of Tet3 and Tet1 or inhibition of BER in hippocampal neurons correlated inversely with excitatory glutamatergic synaptic transmission. Furthermore, Tet3 regulated gene expression in response to global synaptic activity changes and determined glutamate receptor 1 (GluR1) levels at the neuronal surface (Yu et al., 2015). Hence, it was suggested that BER modulates synaptic plasticity and that Tet3 is a sensor to epigenetically regulate the homeostatic synaptic plasticity of neurons. Similarly, a role for the BER protein DNA polymerase β (Polβ) in the early development of postnatal hippocampal pyramidal neurons and learning and memory in mice was recently described in another study. Polβ was implicated as an epigenetic regulator that maintains genome stability in Tet-mediated active DNA demethylation (Uyeda et al., 2020). In this process, the modified base is replaced with cytosine by Polβ. Transgenic mice lacking Polβ expression in forebrain postmitotic excitatory neurons displayed extensive DSBs in hippocampal pyramidal neurons, and to a lesser extent in neocortical neurons. This process was related to the hydroxylation of 5-methylcytosine by Tet1 (Uyeda et al., 2020). Interestingly, hippocampal neurons displaying DNA damage exhibited aberrant gene expression profiles and dendrite formation, but not apoptosis. In adult animals, impaired spatial reference memory and contextual fear memory were detected (Uyeda et al., 2020). Thus, this study links physiological DNA damage to epigenetic regulation and subsequent learning and memory.

Increasing evidence suggests that the introduction and repair of DSBs is associated with long-term memory (LTM) processes. LTM requires the establishment, maintenance, and rearrangement of specific neural networks, within specific brain areas involved in learning and memory. This was recently shown to involve recombination-activating genes (RAGs) that encode components of the protein complex that mediates the rearrangement and recombination of genes of the adaptive immune system (Castro-Perez et al., 2016). V(D)J recombination is a somatic process that occurs in developing lymphocytes during the early stages of T and B cell maturation. It results in the highly diverse collection of immunoglobulins and T cell receptors (TCRs) found in B cells and T cells respectively. This is a tightly regulated process involving the generation of DNA DSBs, and activation of DNA ligases and DNA repair proteins for re-joining the new gene segments. Expression of RAG1 was induced in the amygdala of C57BL/6 mice after context fear conditioning, but not after context-only or shock-only conditioning (Castro-Perez et al., 2016). Furthermore, knock down of RAG1 using antisense oligonucleotides significantly impaired LTM. This study, therefore, implies the existence of a link between RAG1 and DSBs with associative learning processes and in the consolidation of LTMs (Castro-Perez et al., 2016).

**Figure 1:**
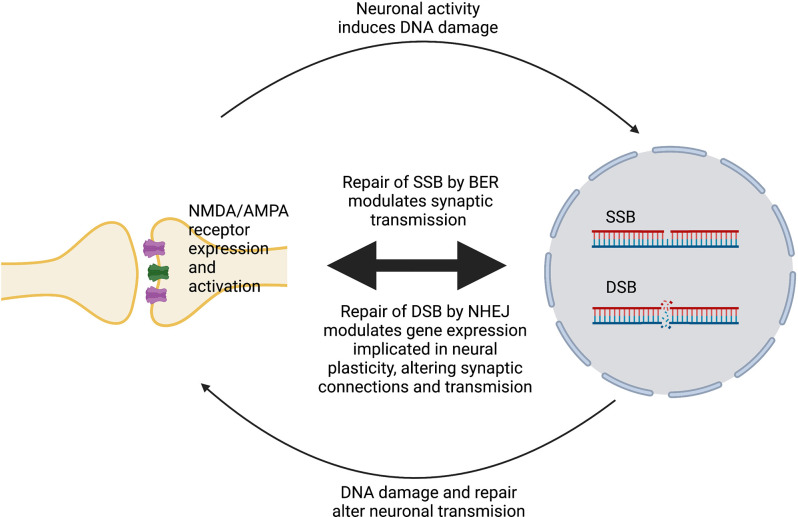
Simplified model of the interplay between neural plasticity and DNA damage and repair. Activation of NMDA/AMPA receptors at synapses induces SSBs or DSBs and promotes their repair by BER or NHEJ, respectively. In turn, DNA damage and repair alters the expression and activity of these receptors, which modulates neuronal gene expression, leading to changes in plasticity.

DNA damage can modulate neural plasticity through the expression of ERGs, which encode transcription factors such as *c-fos*, *c-myc* or *c-jun*. These in turn govern the expression of late response genes such as *bdnf* and *homer1* to regulate various cellular responses. In neurons, ERGs play important roles in cellular, circuitry and cognitive functions, including neurite outgrowth, synapse development and maturation, the balance between excitatory and inhibitory synapses, and learning and memory (Madabhushi et al., 2015). Furthermore, ERGs modulate and maintain neural connectivity in an activity-associated manner (Hogan et al., 2020). Interestingly, regulation of ERG expression is associated with the generation of DSBs (Madabhushi and Kim, 2018) and is triggered by neuronal activation (Madabhushi et al., 2015). Therefore, activity-induced DSBs can trigger transcriptional changes that alter a neuron’s overall generic expression and ultimately induce plasticity changes (Hogan et al., 2020). ERGs are also regulated by a member of the growth arrest and DNA damage (Gadd45) protein family, Gadd45γ, which performs critical roles in the repair of DSBs (Li et al., 2019). Gadd45γ temporally influences learning-induced ERG expression in the prelimbic prefrontal cortex of adult mice through its interaction with DSB-mediated changes in DNA methylation (Li et al., 2019). Another DNA repair protein, ataxia telangiectasia and Rad3-related (ATR), is involved in the regulation of neuronal activity (Kirtay et al., 2021). ATR deletion in neurons enhances intrinsic neuronal activity, resulting in aberrant firing and an increased epileptiform activity, and increased susceptibility to ataxia and epilepsy in mice. ATR-deleted neurons exhibit hyper-excitability, associated with changes in action potential conformation and presynaptic vesicle accumulation, albeit independently of DNA damage response signaling (Kirtay et al., 2021). Figure 1 illustrates a simplified model of the interplay between neuroplasticity and DNA damage and repair. Table 1 summarizes current evidence linking the DDR to neural plasticity.

**Table 1:** Summary of the relationship between the DNA damage response and neural plasticity.

Type of DNA damage	Role in neural plasticity	Implicated DNA repair pathways and/or proteins
SSBs	‐ Modulate the activity of AMPA glutamate receptors (Lu et al., 2001) ‐ Correlate with the expression of NMDA receptor subunits NR1, NR2A, NR2B, and excitatory amino acid transporter 1 (EAAT1; Reus et al., 2015) ‐ Present within enhancers at or near CpG dinucleotides and sites of DNA demethylation (Wu et al., 2021)	‐ BER involving OGG1, NEIL1, (CREB)-mediated APE1 and NF-Kb (Jiang et al., 2003; Wang et al., 2009; Stetler et al., 2010; Yang et al., 2010) ‐ BER-mediated Tet-initiated DNA demethylation and regulation of excitatory glutamatergic synaptic transmission (Yu et al., 2015) ‐ PARP1 and XRCC1 dependent repair of SSBs within enhancers at or near CpG dinucleotides and sites of DNA demethylation (Wu et al., 2021) ‐ ATR regulates neuronal activity (Kirtay et al., 2021)
DSBs	‐ Correlate with expression of NMDA receptor subunits NR1, NR2A, NR2B, and excitatory amino acid transporter 1 (EAAT1; Reus et al., 2015) ‐ Generated in response to contextual fear conditioning (Stott et al., 2021) ‐ Modulate expression of ERGs such as c-fos, c-myc, c-jun (Madabhushi et al., 2015)	‐ Mre11–dependent DNA repair upon activation of ionotropic NMDA or AMPA/kainite glutamate receptors (Crowe et al., 2006) ‐ NHEJ putatively repairs DSBs within ERG promoters induced by neuronal activity (Madabhushi et al., 2015) ‐ BER-mediated Polβ and Tet prevent DSBs formation (Uyeda et al., 2020)

## Physical Activity Is Associated with DNA Damage and Plasticity

Physical activity induces neuronal stimulation, resulting in genetic alterations that regulate synaptic and brain plasticity (Vilela et al., 2020). Chronic exercise has a positive response on the brain, which is different compared with other organs (Liu et al., 2000). Aerobic and strength training improves spatial memory, reduces DNA damage, and increases expression of brain-derived neurotrophic factor (BDNF) and CREB in aging rats (Kim et al., 2010; Vilela et al., 2017) and mice (Yang et al., 2014). Physical exercise promotes DNA repair by activation of the enzymes involved in BER repair in human and animal models (Radak et al., 2009; Koltai et al., 2010; Siu et al., 2011). Regular exercise attenuates the age-associated increase in 8-hydroxy-2’-deoxyguanosine levels and increases DNA repair activity and neuronal resistance against oxidative stress (Radak et al., 2002). Similarly, an increase in the activity of 8-oxoG in human skeletal muscle was detected after a single-bout of physical exercise (Radak et al., 2003). In contrast, strenuous endurance exercise can result in muscle and lymphocyte DNA damage and the extent of this increases as running distance increases (Ryu et al., 2016).

The studies described above refer to the role of DNA damage in the normal physiological function of neurons. Below we describe the relationship between DNA damage and plasticity in relation to neurodegenerative diseases.

## DNA Damage and Plasticity in Neurodegeneration

DSBs can alter gene expression, chromatin stability, and cellular functions, hence they can be an important driver of neurodegeneration and cognitive decline in neurodegenerative diseases. Aging is the major risk factor for neurodegenerative diseases. Interestingly, with aging the expression of genes related to synaptic plasticity and DNA repair is reduced (Lu et al., 2004). Moreover, DNA damage is increased in the promoters of genes that display reduced expression in the aged human cortex. Furthermore, these genes are selectively damaged by oxidative stress in cultured human neurons, resulting in reduced BER repair. These findings imply that DNA damage may reduce the expression of selectively vulnerable genes involved in learning and memory (Lu et al., 2004). Here we review the evidence linking dysfunction of the DDR with neural plasticity in the diseases described below.

### Alzheimer’s Disease (AD)

Alzheimer’s disease (AD) is a neurodegenerative disorder resulting in brain atrophy and death of neurons located primarily in the hippocampus and cerebral neocortex. AD is the most common cause of dementia, which manifests as a continuous decline in thinking, behavioral and social skills, impairing person’s ability to function independently. Two hallmark pathologies are present in AD; both β-amyloid plaques and neurofibrillary tangles, containing hyperphosphorylated tau (Weller and Budson, 2018). The accumulation of DSBs is associated with loss of memory and neurons in AD (Lu et al., 2004; Shanbhag et al., 2019; Lin et al., 2020) and impairment of DNA repair processes is present prior to the onset of symptoms (Su et al., 1997; Sheng et al., 1998; Silva et al., 2014). More _γ_H2AX foci, a widely used marker of DSBs, were present in neurons and astrocytes in the hippocampus and frontal cortex of AD patients compared to age-matched controls (Shanbhag et al., 2019). In this study some neurons and glia displayed diffuse pan-nuclear patterns of _γ_H2AX immunoreactivity, which was not associated with DSBs, suggesting that DSBs accumulate selectively in vulnerable neuronal and glial cell populations (Shanbhag et al., 2019). Interestingly, β-amyloid inhibits DNA-PK-dependent NHEJ and contributes to the accumulation of DSBs in PC12 cells (Cardinale et al., 2012). Furthermore, whilst DSBs are generated in the dentate gyrus of wildtype mice in response to the exploration of a novel environment, they are repaired within 24 h (Suberbielle et al., 2013). In contrast, an AD transgenic mouse model expressing human amyloid precursor protein (hAPP) with aberrant neuronal activity, displayed more neuronal DSBs before and more severe and prolonged DSBs after, exploration of a novel environment. This finding suggests that excessive accumulation of DSBs in AD impairs spatial learning and memory. Interestingly, suppression of aberrant neuronal activity by ablating expression of tau, improved memory and led to the production of fewer DSBs in this model, suggesting that excessive DNA damage in AD is associated with synaptic dysfunction (Suberbielle et al., 2013). Furthermore, evidence for loss of function of ATM was detected in three different AD transgenic mouse models; R1.40, which expresses a single (full-length) APP transgene, PS/APP, expressing transgenes for both APP and presenilin-1 (PSEN1) and triple-transgenic animals (3xTg), which bear APP, PSEN1, and MAPT transgenes, together implying that defective repair of DSBs is present in AD (Shen et al., 2016). Excessive accumulation of DSBs in AD has also been linked to impairment of HR repair. In PS/APP mice, more persistent DSBs were present compared to control animals. This was accompanied by diminished HR repair in the subgranular zone of the dentate gyrus, the site of neurogenesis (Yu et al., 2018). In addition, disturbance in the cell cycle and DNA damage was present in neurons reprogrammed from induced pluripotent stem cells (iPSC) derived from familial AD patients. Specifically, an increase in a component of HR repair, a human tumor suppressor, BRACA1, was reported (Wezyk et al., 2018). BER repair is also linked to aberrant neural plasticity in AD. A significant decrease in NEIL DNA glycosylase was detected in AD brains compared to controls (Canugovi et al., 2014). Interestingly, NEIL1 null mice display defective memory retention in a water maze test (Canugovi et al., 2012). Furthermore, 3xTgAD mice with inefficient BER due to DNA polymerase-β (Polβ) haploinsufficiency displayed enhanced neurodegeneration, synaptic and cognitive deficits, and more DNA damage, compared to 3xTgAD mice with normal levels of Polβ (Sykora et al., 2015). Furthermore, olfactory deficits are present early in AD and these 3xTgAD/ POLβ+/− mice display greater degeneration of olfactory bulb neurons, in part *via* inhibiting the production of new neurons from neural progenitor cells (Misiak et al., 2017). Similarly, increased oxidative DNA damage and deficient BER was detected in post-mortem brain tissue samples derived from patients with mild cognitive impairment and AD, compared to age-matched neurologically normal subjects (Weissman et al., 2007). Consistent with this finding, reduced 8-oxoG activity was present in the hippocampal and para-hippocampal gyri, superior and middle temporal gyri, and inferior parietal lobule in AD patients compared to control individuals. This decrease was accompanied by altered DNA helicase activity, suggesting that this function may interfere with BER repair in AD (Lovell et al., 2000).

The deficiency of Polβ and decreased levels of NAD+, a cellular metabolite critical for neuronal resistance to stress and maintenance of DNA integrity, were found in AD patients’ brains (Hou et al., 2018). In an AD mouse model with deficiencies in DNA repair, NAD+ supplementation improved the DNA damage response and synaptic transmission, learning and memory, and motor function (Hou et al., 2018). Together these findings highlight the importance of efficient DNA repair processes in neuronal function related to neural plasticity in AD (Hou et al., 2018).

### Amyotrophic Lateral Sclerosis (ALS)

Amyotrophic lateral sclerosis (ALS) is a motor neuron disorder characterized by progressive loss of motor neurons in the cortex, brainstem, and spinal cord. This leads to muscle atrophy and weakness, eventually resulting in death. ALS overlaps significantly with frontotemporal dementia (FTD), the most common form of early-onset dementia (under 60 years of age) that primarily affects the frontal and temporal lobes of the brain (Shahheydari et al., 2017). DNA damage is increasingly implicated in the pathophysiology of ALS, and interestingly, the number of DNA repair proteins linked to ALS is steadily growing. Similarly, disruption of neurotransmission is associated with ALS pathogenesis. Cortical hyperexcitability, the enhanced response of a neuron to a stimulus, is an important pathophysiological process in ALS, contributing to the loss of motor neurons (Buskila et al., 2019; Brunet et al., 2020). Studies on ALS patients have revealed that this precedes clinical symptoms (Vucic and Kiernan, 2006), contributes to disease progression, and correlates with greater functional disabilities (Menon et al., 2020). Whilst the underlying mechanisms are unknown, hyperexcitability is related to the interplay between excitatory and inhibitory interneurons (Bae et al., 2013). Excitotoxicity involving the neurotransmitter glutamate, hypo-excitability, and loss of repetitive firing are also present in vulnerable motor neurons *in vivo* (Foran and Trotti, 2009; Martinez-Silva et al., 2018). Furthermore, frequent and strenuous physical activity is implicated as a risk factor for ALS (Julian et al., 2021). Importantly, this is known to induce DNA damage and is also related to changes in synaptic plasticity (Ryu et al., 2016; Vilela et al., 2020).

Interestingly, proteins that are central to ALS pathogenesis are implicated in both DNA damage and plasticity. Pathological forms of TAR DNA-binding protein 43 (TDP-43), a heterogenous nuclear ribonucleoprotein (hnRNP), is present in affected motor neurons in almost all ALS cases (97%). TDP-43 modulates RNA splicing and micro-RNA biogenesis and it also functions in NHEJ DNA repair (Mitra et al., 2019; Konopka et al., 2020). Its pathological mis-localization to the cytoplasm of motor neurons and the presence of ALS-associated mutations in TDP-43 are both known to impair DNA repair (Mitra et al., 2019; Konopka et al., 2020). TDP-43 also regulates synaptic functions. Transgenic animals expressing human A315T mutant TDP-43 exhibit reduced levels of the presynaptic protein synaptophysin in the brain, and display attenuated synaptic transmission, cognitive and motor deficits (Medina et al., 2014). TDP-43 depletion in transgenic rats enhances the acquisition of fear memory, decreases the short-term plasticity of intrinsic neuronal excitability, and affects the kinetics of AMPA receptors by slowing the decay time of AMPAR-mediated miniature excitatory postsynaptic currents (Koza et al., 2019). These findings imply that TDP-43 regulates activity-dependent neuronal plasticity, possibly by controlling the splicing of genes responsible for fast synaptic transmission and membrane potential (Koza et al., 2019). Interestingly, DNA damage modifies splicing proteins by modulating their activity or interaction with other proteins or RNA (Shkreta and Chabot, 2015), linking the role of TDP-43 in DNA damage with its role in activity-dependent neuronal plasticity.

Fused in sarcoma (FUS) is another DNA/RNA-binding protein implicated in both DNA repair and synaptic dysfunction in ALS, that displays structural and functional similarities to TDP-43. FUS interacts with histone deacetylase 1 (HDAC1) to facilitate DNA repair through NHEJ, while FUS-ALS associated mutants R514S and R521C display impaired DNA repair functions (Wang et al., 2013). FUS-ALS associated mutants R521C and P525L disrupt the formation of presynaptic active zones, subsequently reducing synaptic transmission with decreased quantal size (Machamer et al., 2014). Hence these studies imply a relationship between DNA repair and synaptic activity in ALS.

The DNA damage response is also induced by hexanucleotide (GGGGCC) repeat expansions in a non-coding region of C9orf72, the major genetic cause of ALS (Farg et al., 2017). In *Drosophila*, transgenic expression of the C9orf72 repeat expansion results in a dramatic reduction in synaptic arborization and the number of active zones at neuromuscular junctions (NMJs), and reduced neurotransmission. Hence synaptic dysfunction at NMJs is induced by C9orf72 ALS related pathology (Perry et al., 2017). Another prominent protein linked to familial forms of ALS, superoxide dismutase-1 (SOD1), is also implicated in both synaptic dysfunction and DNA damage. SOD1 localizes at the pre- and post-synapse, while the ALS-associated mutant G93A SOD1 shows mis-localization in pre-synaptic terminals as well as at the post-synapse, impairing axonal transport and contributing to neuronal cell death (Lee et al., 2015; Bae and Kim, 2016). Expression of mutant SOD1 G93A also decreases the formation of synaptophysin-positive presynaptic boutons (Zang et al., 2005). In ALS, over-activation of glutamate receptors in motor neurons is a well-described pathological event (Corona et al., 2007). Motor neurons from transgenic SOD1 G93A mice are sensitive to glutamate toxicity and this was associated with ROS production, resulting in oxidative DNA damage, elevation in intracellular calcium levels, and mitochondrial dysfunction (Kruman et al., 1999). Substantial increases in oxidative DNA damage have also been observed in nuclear DNA from the spinal cord, frontal cortex, striatum, and cerebellum from the transgenic G93A SOD1 mouse model (Aguirre et al., 2005). Therefore, taken together, these findings indicate that dysfunctional DNA repair and dysfunctional neural plasticity occur together in ALS.

### Huntington’s Disease (HD)

Huntington’s disease (HD) is an inherited autosomal dominant neurodegenerative disorder characterized by motor dysfunction and cognitive deficits, due to neurodegeneration of specific brain regions such as the striatum and cerebral cortex. It is caused by mutation of the huntingtin protein, resulting in an abnormally high copy of polyglutamine (polyQ) repeats at its N-terminus. The expression of mutated huntingtin enhances NMDA activity and sensitizes type 1 inositol 1,4,5-trisphosphate receptors, causing a disturbance in calcium homeostasis (Bezprozvanny and Hayden, 2004; Zhang et al., 2008). In addition, expression of the presynaptic protein rabphilin 3A is decreased (Deak et al., 2006), while the levels of synaptic vesicle protein SCAMP5 are increased, in *Caenorhabditis elegans* neurons (Parker et al., 2007). This implies that alteration of expression of presynaptic proteins results in impairment of synaptic vesicle fusion or endocytosis. HD is also associated with DNA damage and defective DNA repair. Post-mortem brain samples of HD patients display higher levels of the oxidative DNA damage marker 8-Oxo-dG in both nuclei and mitochondria (Browne et al., 1997; Polidori et al., 1999). In addition, mutant huntingtin impairs NHEJ DNA repair by interaction with Ku70 protein, leading to the accumulation of DSBs in primary neurons (Enokido et al., 2010). Interestingly, expression of exogenous Ku70 rescues abnormal behavior and pathological phenotypes in the R6/2 mouse model of HD (Enokido et al., 2010). These findings imply that the huntingtin protein has a dual role in synaptic plasticity and DNA repair. For comprehensive reviews about the role of DNA damage in HD, we direct readers to the following articles about the role of mitochondrial DNA damage in HD (Yang et al., 2008), the role of MMR in HD (Iyer and Pluciennik, 2021), and the role of HR in HD (Jeon et al., 2012).

### Parkinson’s Disease (PD)

Parkinson’s disease (PD) is a neurodegenerative disorder associated with widespread degradation of dopaminergic neurons in the substantia nigra *pars compacta* (SNc), with subsequent loss of the neurons projecting to the striatum (Lang and Lozano, 1998). PD manifests principally by motor symptoms such as bradykinesia, rigidity, tremor at rest, postural instability, micrographia, and shuffling gait (Jankovic, 2008). Familial Parkinsonism is linked to mutations in a number of genes including α-synuclein, LRRK2, parkin, DJ-1, PINK1, UCHL-1, synphilin-1, and NR4A2 (Nussbaum and Polymeropoulos, 1997; Mizuno et al., 2001; Le et al., 2003; Marx et al., 2003; Healy et al., 2004; Maraganore et al., 2004; Valente et al., 2004; Rui et al., 2018).

Both DNA damage and dysfunction of synaptic plasticity have been reported in PD. Altered presynaptic plasticity is present in PD animal models, including α-synuclein, DJ-1, and PINK1 knock out mice (Abeliovich et al., 2000; Goldberg et al., 2005; Kitada et al., 2007). Similarly, altered vesicular transmission and defects in neurotransmission are present in experimental Parkinsonism (Abeliovich et al., 2000; Murphy et al., 2000; Li et al., 2010). Furthermore, altered composition of glutamatergic NMDA receptors contributes to clinical features of experimental Parkinsonism (Ulas and Cotman, 1996; Dunah and Standaert, 2001; see Picconi et al., 2012 for a comprehensive review about dysfunction to synaptic plasticity in PD). Similar to dysfunctional plasticity, DNA damage is present in the SNc of PD patients, including elevated levels of 8-oxo-guanine, abasic sites, and nuclear DNA strand breaks (Alam et al., 1997; Zhang et al., 1999; Hegde et al., 2006; Sanders et al., 2014; see Gonzalez-Hunt and Sanders, 2021 for a comprehensive review of DNA damage in PD). α-synuclein, which is known to function in synaptic plasticity, is also linked to DNA damage in PD. Overexpression of α-synuclein causes the formation of SSBs and DSBs, particularly under oxidative conditions (Vasquez et al., 2017) and it modulates repair of DSBs (Schaser et al., 2019). Depletion of Parkin results in impairment of dopamine release and synaptic plasticity in the striatum of Parkin knockout mice (Kitada et al., 2009). Interestingly, mutations in Parkin also result in the accumulation of BER factor, APE1 (Scott et al., 2017), suggesting that defective Parkin causes dysfunction to both DNA repair and synaptic plasticity. Mice with mutated excision repair cross-complementation group 1 (ERCC1) endonuclease, a competent of the NER repair pathway, display PD pathology, including impairment of striatal innervation, α-synuclein pathology, and the formation of _γ_H2AX foci (Sepe et al., 2016). This implies an important role for DNA repair pathways in integrity of the nigrostriatal system in PD. Table 2 illustrates the potential and/or established roles of mutant proteins associated with neurodegeneration in DNA damage response mechanisms.

**Table 2:** Summary of the DNA damage and repair proteins associated with neural plasticity in neurodegenerative diseases.

Disease	Known disease proteins with potential/established role in DNA repair	DNA repair proteins not specifically linked to the disease	Impact on neuroplastic processes
AD		Reduced expression of NEIL DNA glycosylase, implying impairment of BER (Canugovi et al., 2014)	‐ Impairment of memory retention (Canugovi et al., 2012, 2014)
		Reduced expression of Polβ, implying impairment of BER (Sykora et al., 2015)	‐ Impairment of memory and synaptic plasticity (Sykora et al., 2015) ‐ Olfactory deficits (Misiak et al., 2017) ‐ Attenuation of generation of new neurons by neural progenitor cells (Misiak et al., 2017)
		Loss of function of ATM (Shen et al., 2016)	‐ Loss of memory (Shen et al., 2016; Yu et al., 2018) ‐ Aberrant neuronal activity (Shen et al., 2016; Yu et al., 2018) ‐ Synaptic dysfunction (Shen et al., 2016; Yu et al., 2018)
		Reduced expression of RAD51 (Yu et al., 2018)
ALS	TDP-43 functions in NHEJ, and is mutated and forms pathological aggregates in ALS (Mitra et al., 2019; Konopka et al., 2020)		‐ Reduced synaptophysin (Medina et al., 2014) ‐ Attenuated synaptic transmission (Medina et al., 2014) ‐ Cognitive and motor deficits (Medina et al., 2014) ‐ Decreased short-term plasticity (Koza et al., 2019) ‐ Altered kinetics of AMPA receptors (Koza et al., 2019)
	FUS functions in NHEJ and BER, and is mutated and forms pathological aggregates in ALS (Wang et al., 2013)		‐ Disrupted formation of presynaptic active zones (Machamer et al., 2014) ‐ Reduced synaptic transcription (Machamer et al., 2014)
	C9orf72 is mutated in ALS, and expression of the repeat expansion induces DNA damage (Farg et al., 2017)		‐ Reduction in synaptic arborization (Perry et al., 2017) ‐ Dysfunctional neuromuscular junctions (Perry et al., 2017) ‐ Reduced neurotransmission (Perry et al., 2017)
	SOD1 is mutated in ALS, which induce DNA damage (Lee et al., 2015; Bae and Kim, 2016)		‐ Pre and post synaptic localization (Zang et al., 2005; Lee et al., 2015; Bae and Kim, 2016) ‐ Over-activation of glutamate receptors (Zang et al., 2005; Lee et al., 2015; Bae and Kim, 2016)
HD	Huntingtin functions in DSB repair and is mutated in HD (Bezprozvanny and Hayden, 2004; Zhang et al., 2008; Enokido et al., 2010)		‐ Enhanced NMDA activity (Parker et al., 2007) ‐ Impairment of synaptic vesicle fusion or endocytosis (Parker et al., 2007)
PD	α-synuclein modulates repair of DSBs and is mutated in PD (Abeliovich et al., 2000; Schaser et al., 2019)		‐ Altered presynaptic plasticity (Abeliovich et al., 2000)
		ERCC1 endonuclease—a component of NER repair (Sepe et al., 2016)	‐ Alternation in striatal innervation (Sepe et al., 2016)

## Conclusions

Neurons, in contrast to other cell types, possess unique features, including their ability to undergo electrical excitation and their post-mitotic character. Recent findings imply that DNA damage is not limited to pathological conditions as previously thought, but it is also important for unique neuronal functions related to neural and synaptic plasticity under physiological conditions. However, dysfunction in these processes is also related to a decline in cognitive function and neuronal death in neurodegenerative diseases. However, human post-mortem tissues represent the end-point stage of the disease. Hence studies examining these tissues cannot be used to determine whether DNA damage has a primary or secondary role in pathogenesis. However, future studies on the relationship between plasticity and DNA damage may provide a better understanding of the cellular processes that contribute to higher order brain functions. Distinct groups of neurons are affected in different neurodegenerative diseases, such as motor neurons in ALS or neurons of the entorhinal cortex in AD, and these cells are specialized to perform specific functions. Given that DNA damage and repair are important for the unique functions of neurons, which in turn depend on their activation, it is possible that the interplay between DNA damage and neural plasticity is unique for specific groups of neurons. This could operate through the activation of specific genes by DNA damage, which would differ depending on the type of neurons involved and their associated functions. Therefore, a better understanding of the interplay between DNA damage and neural plasticity is required, as well as dysfunction in these processes in disease. In particular, the inclusion of specific neuronal types may reveal the causes of selective neuronal death in distinct neurodegenerative diseases. To date, no previous studies have examined therapeutic strategies directed at DNA damage and repair in relation to aberrant neural plasticity. However, this approach has the potential to identify novel treatments for impaired cognitive functions in neurodegenerative diseases associated with excessive DNA damage.

## Author Contributions

AK conceptualized and wrote the manuscript. AK provided resources for the figure preparation, and prepared the figure and tables. JA provided all other resources, and contributed with intellectual input and additional text. AK and JA edited the manuscript and approved it for publication. Both authors contributed to the article and approved the submitted version.

## Conflict of Interest

The authors declare that the research was conducted in the absence of any commercial or financial relationships that could be construed as a potential conflict of interest.

## Publisher’s Note

All claims expressed in this article are solely those of the authors and do not necessarily represent those of their affiliated organizations, or those of the publisher, the editors and the reviewers. Any product that may be evaluated in this article, or claim that may be made by its manufacturer, is not guaranteed or endorsed by the publisher.

## Footnotes

^1^ https://www.nature.com/collections/hwnqqcstyj/
